# Climate change and bird reproduction: warmer springs benefit breeding success in boreal forest grouse

**DOI:** 10.1098/rspb.2017.1528

**Published:** 2017-11-08

**Authors:** Per Wegge, Jørund Rolstad

**Affiliations:** 1 Faculty of Environmental Sciences and Natural Resource Management, Norwegian University of Life Sciences, PO Box 5003 NMBU, 1432, Ås, Norway; 2 Department of Forest Genetics and Biodiversity, Norwegian Institute of Bioeconomy Research, PO Box 115, 1431, Ås, Norway

**Keywords:** black grouse, breeding, capercaillie, climate change, global warming, grouse reproduction

## Abstract

Global warming is predicted to adversely affect the reproduction of birds, especially in northern latitudes. A recent study in Finland inferred that declining populations of black grouse, *Tetrao tetrix*, could be attributed to advancement of the time of mating and chicks hatching too early—supporting the mismatch hypothesis. Here, we examine the breeding success of sympatric capercaillie, *T. urogallus,* and black grouse over a 38-year period in southeast Norway. Breeding season temperatures increased, being most pronounced in April. Although the onset of spring advanced nearly three weeks, the peak of mating advanced only 4–5 days. In contrast to the result of the Finnish study, breeding success increased markedly in both species (capercaillie: 62%, black grouse: 38%). Both brood frequency and brood size increased during the study period, but significantly so only for brood frequency in capercaillie. Whereas the frequency of capercaillie broods was positively affected by rising temperatures, especially during the pre-hatching period, this was not the case in black grouse. Brood size, on the other hand, increased with increasing post-hatching temperatures in both species. Contrary to the prediction that global warming will adversely affect reproduction in boreal forest grouse, our study shows that breeding success was enhanced in warmer springs.

## 1. Introduction

A warmer climate is predicted to have pronounced impacts on local fauna and flora [1,2], especially in northern latitudes [3]. The breeding performance of many birds is likely to be adversely affected [4–7]. More than a decade ago, Visser *et al.* [8] predicted that warmer springs should lead to earlier mating and a temporal mismatch with the optimal hatching time for the offspring. In a later study of the breeding phenology of an insectivorous passerine, the great tit (*Parus major*), they showed that the optimal synchrony between hatching time and irruption of caterpillar food supply was distorted in years with earlier and warmer springs [9]. Later studies have largely confirmed this ‘mismatch’ hypothesis both for non-migratory [10] and migratory birds [11].

Until recently, studies of the effects of climate change on non-migratory birds have mainly dealt with passerines. In the temperate zone, only four studies have reported on the larger tetraonids. In Finland, Ludwig *et al.* [12] modelled the temperature and timing of mating (and subsequent hatching) of black grouse, *Tetrao tetrix,* from time-series data and showed that earlier mating in later years correlated with increasing temperatures during April and May. As temperature during hatching in June decreased slightly, they inferred that earlier hatching would increase the mortality of chicks because they would then be born in colder weather and with less food for the hatchlings. They concluded that the asymmetrical rise in temperature and mismatch in breeding phenology probably explained the ongoing decline in black grouse in the country. However, a later study of capercaillie, *Tetrao urogallus,* in Norway reported that during a year of early breeding, more fledged chicks were produced although the staple chick food source (insect larvae) did not advance [13].

A different scenario of climate change leading to reduced breeding success (i.e. number of chicks reared per female) in forest grouse was launched by Selås *et al*. [14] from Norway: following peak seed years of bilberry, *Vaccinium myrtillus*—a main food plant and host of caterpillar food for the chicks—breeding success should be enhanced due to reduced feeding deterrents in the plants. However, in years with particularly high summer temperatures, bilberry will be less exhausted and rebuild their chemical defence quickly, therefore leading to poorer breeding the following year. They examined weather records and time-series data on abundances of capercaillie and black grouse and reported an inverse relationship with high summer temperatures during 1 or 2 years before peak bilberry seed production. From this, they predicted that warmer summers would limit grouse reproduction.

A third study from Scotland [15] reported that breeding success in capercaillie declined during 1975–1999 and found evidence that this might be related to a gradual delay in April warming due to a short cooling period in the middle of that month. They speculated that the sudden burst of nutritious new plant growth that had previously accompanied an abrupt increase in spring temperature became slower and more erratic. Slower-growing plant food is less nutritious and so the hens' diet became less nutritious, and their eggs and chicks less viable. Lastly, breeding performance of black grouse with climate change has been investigated by Barnagaud *et al*. [16] in the French Alps. During 1990–2007, no relationship was detected between breeding success and the winter and summer North Atlantic Oscillations.

In a summary article from a long-term study of the population ecology of capercaillie and black grouse in our study area (Varaldskogen, southeast Norway), we reported that the breeding success of both capercaillie and black grouse increased gradually during a 30-year period from 1979 to 2009 [17]. In view of the current focus on the possible negative effects of warmer springs, we here examine the relationship between spring and early summer temperatures and breeding success from this population study in more detail. The results related to the timing of breeding and chick food cited above [13] were derived from the same study but based on data in only two contrasting years. Expanding the time series to 38 years, we now confirm that breeding success was higher—not lower—in warmer springs.

## 2. Study area

Located next to the Swedish border in southeast Norway (60°10′ N, 12°30′ E), the study area covers about 100 km^2^, of which the 40 km^2^ eastern part of the Varald State Forest constituted an intensively monitored core area. The gently undulating terrain between 200 and 400 m a.s.l. consists of Norway spruce *Picea abies* and Scots pine *Pinus silvestris* interspersed with scattered birch *Betula* spp. and aspen *Populus tremula*. A widely distributed dwarf shrub in the field layer is bilberry *Vaccinium myrtillus,* an important food plant of forest grouse, including newly hatched broods. The ground-nesting black grouse and capercaillie are large (approximately 1–3 kg live weight, respectively), ground-nesting birds which hatch their 7–9 precocial and nidifugous chicks in early June. Owing to poorly developed thermoregulation during 3–4 days after hatching, the chicks are quite vulnerable to cold weather [18], and geometrid and sawfly caterpillars are their main food during their first weeks of life [19–22]. In the study area, the two species occur at moderate densities of 2.5–3 birds km^−2^ in spring, together with a few and patchily distributed hazel grouse, *Bonasa bonasia*. Main predators are red fox, *Vulpes vulpes*, pine marten, *Martes martes*, goshawk, *Accipiter gentilis*, and buzzard, *Buteo buteo*; other raptors and mammalian predators are seasonal visitors or quite rare.

Contiguous with other mixed coniferous forests on all sides—only interspersed with small patches of small abandoned farmlands—the forest has been subjected to timber harvesting for several centuries. Notably, in the 1950s, clear-cutting became the main harvesting regime, replacing the traditional high-grading and selection methods. Cutting blocks were initially rather large (more than 50 ha in the 1960s–1970s), but have successively been reduced in size, today rarely exceeding 20 ha. During the last four decades, the coverage of semi-natural old coniferous forests has decreased to less than 20%, the remaining consisting of middle-aged and young silvicultural stands of pure and mixed plantations of spruce and pine. For more details, see Wegge & Rolstad [17].

## 3. Material, methods, and analyses

The abundance and sex/age composition of capercaillie and black grouse were sampled each August by teams using trained pointing dogs; a method commonly used for censusing tetraonids [15,17,23]. The study area was subdivided into 42 sampling blocks, within which observers with dogs searched for birds. Rarely was the whole block sampled thoroughly enough for converting the number of birds encountered to a density estimate. Instead, the number of different bird categories was expressed as numbers/10 h of sampling time. A random selection of blocks was sampled each year, covering at least 60% of the study area. During sampling, each man/dog team was equipped with a map of the block, and a form on which to record encountered birds and time of observation. On the map, the corresponding locations and flight directions were recorded; this was done in order to minimize double counts of birds within the same block or of birds flying into neighbouring blocks sampled the same day. Altogether, between 112 and 186 birds were recorded and classified each year, totalling nearly 6 000 observations over the 38-year period.

Capercaillie and black grouse are both polygynous, lekking species, with females visiting the leks during 5–10 days in late April and early May [13,24]. In capercaillie, most females mate over the course of 3–4 days, whereas for black grouse leks, mating takes place over a longer period, peaking 4–7 days later than in capercaillie in our study area (unpublished material). Each year, we sampled 4–6 capercaillie leks and recorded the dates of female attendance and mating, from which we could estimate the peak of mating. From the known time of mating and hatching of 23 radio-collared females, we could then estimate the average number of days from mating until hatching in this species. Black grouse leks were not systematically monitored; although male counts were made on 3–4 leks each year, female attendance and mating were recorded only intermittently. For this species, the annual date of hatching was estimated from the monitoring of 12 radio-collared females and from observed differences in hatching dates in the two species in the same year [13]. Based on these data, we have used 5 days later date of mating and 6 days later date of hatching in black grouse compared to capercaillie throughout the study period.

Breeding success is the outcome of mainly two life-history traits, the loss of eggs plus the loss of chicks. In addition, a variable but small number of females do not attempt breeding or desert their nests, and some eggs do not hatch; in capercaillie the proportion of non-breeders is thought to be higher than in black grouse, presumably because some yearling females do not breed. In August, brood frequency (i.e. the proportion of females with at least one chick) therefore largely reflects the rate of egg loss, whereas brood size (i.e. the number of chicks per brood) indicates closely the rate of chick loss after hatching. Hence, their relative proportions of the breeding success before and after hatching point to the causal factors in these two periods of the breeding season. We therefore examined these two components separately in the analyses.

In Fennoscandia, predation on eggs and chicks varies with cyclic small rodents—the so-called alternative prey hypothesis [25,26]. In years with high abundance of small rodents, predators—mainly red fox and pine marten—prey and subsist mainly on this food source, whereas in crash years they switch to other food, of which grouse eggs and chicks make up a large proportion. This temporal variation in predation pressure has been shown to be an important determinant of breeding success in forest grouse [27–29]. Hence, we included information on changes in abundances of small rodents and red fox in the analyses. Rodents were sampled each year in the study area [17], and indices of fox abundance were taken from regional harvest data, adjusted to our study area based on snow-tracking indices in winters with snow cover.

Weather variables included daily minimum, average and maximum temperatures, precipitation, and the amount of snow cover. Data were obtained from Kongsvinger meteorological station, located 25 km from the study area. With our study area situated at a slightly higher elevation, local differences were adjusted for by applying a lapse rate of 0.65-degree change per 100 m difference in elevation. We used two variables to indicate the phenological onset of spring: the date of snow-free ground and the date of frost-free nights. The first was defined as the earliest date that the meteorological station reported less than 20% snow cover (score 0 on a scale from 0–5). The latter was calculated as the date when the 7-day moving average of the daily minimum temperature crossed 0°C. To indicate the temporal onset of summer, we looked at the cumulative temperature sum. Growing degree days (GDD) is defined as the number of temperature degree days above a certain threshold base temperature. Here we used a base temperature of 5°C and defined the onset of summer as the date when GDD reached 200 degree days, which in our study area closely matched the date when the growth of the new shoots and leaves of bilberry is completed (P Wegge 2017, unpublished data).

From the daily spring and summer temperature series, we derived monthly means (April, May, and June) and means for 13 successive time periods throughout the breeding season. Long-term trends were compared using generalized least-squares (GLS) regressions with and without AR(1) and AR(2) correlation structure. Overall, autocorrelation was low and statistically non-significant with Akaike Information Criterion (AIC) values similar or lower for models without autoregressive corrections. Throughout the results, we therefore report ordinary least square regression coefficients (*β*) with corresponding standard errors or correlation coefficients (*r*). When testing for relationships between breeding success and weather variables on an annual basis, autocorrelation was checked for using an exponential spatial correlation structure (corExp; nlme package of R; [30]). As no significant autocorrelation was detected, we also here report statistics of ordinary least square regressions. We used multiple regression to separate the contributions of temperature and other influential variables (e.g. rodents, fox, precipitation etc.). Right-skewed variables (red fox and rodents) were transformed using log- or square-root values.

## 4. Results

**Figure 1.**
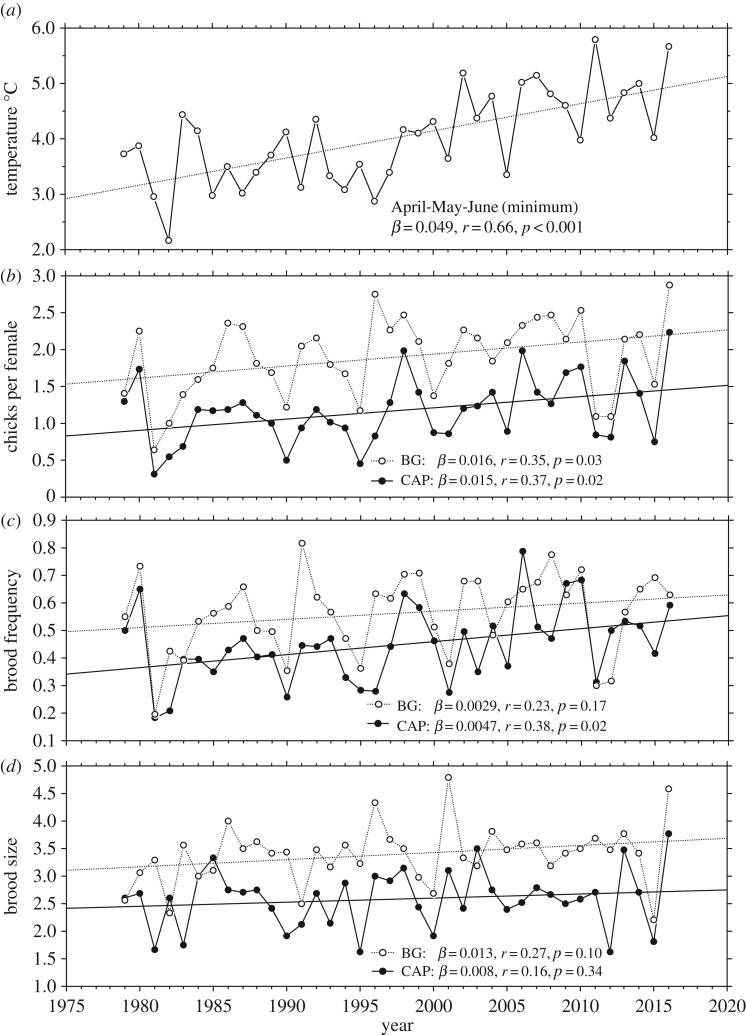
(*a*) April–June averages of minimum daily temperatures, (*b*) breeding success (chicks per female), (*c*) brood frequency (females with broods), and (*d*) brood size in capercaillie (CAP) and black grouse (BG) during 1979–2016. Trend lines are ordinary least squares (OLS)-fitted regression lines, with accompanying slopes (*β*) and correlation coefficients (*r*).

The breeding success of both capercaillie and black grouse increased markedly during the 38-year period, with an absolute increase of 0.57 and 0.62 chicks per female, respectively (figure 1*b*). Owing to overall lower breeding success in capercaillie, the proportional trend appeared to rise steeper than in black grouse (62 versus 38%). However, due to large year-to-year variation, the relative difference between the two species was not statistically significant (log-ratio *β* = 0.0019, *t* = 1.21, *p* = 0.23). The two demographic traits that determine breeding success—brood frequency and brood size—both displayed upward trends during the study period, although this was statistically significant only for brood frequency in capercaillie (figure 1*c*,*d*). In relative terms, brood frequency appeared to have increased more than brood size in capercaillie (normalized *β* of 0.034 versus 0.014), but the difference was not significant (*t* = 1.00, *p* = 0.32). In black grouse, the two traits contributed roughly equally to the overall increase in breeding success (normalized *β* of 0.020 versus 0.024).

Overall, the temperature during the whole breeding season (April, May, and June combined) increased about two degrees throughout the 38-year period (figure 1*a*). The temperature change was most pronounced in April (the pre-incubation period), when the minimum, average, and maximum temperatures rose 1.9, 2.6, and 3.8 degrees, respectively. In June, the temperature changed less, and only the minimum temperature increased more than 1 degree (table 1). The minimum, average, and maximum daily temperatures were highly cross-correlated, but minimum daily temperature correlated best with breeding success in both species. The minimum daily temperature also had the smallest year-to-year variation (standard errors, table 1); hence, we compared breeding performance with the change in minimum temperature in the subsequent analyses.

**Table 1.** Yearly variation and long-term trends (Δ) in temperatures of April, May, and June, and during periods of four weeks pre-incubation, during incubation, and during four weeks post-hatching in capercaillie and black grouse during 1979–2016.

	temperature, daily minimum					temperature, daily average					temperature, daily maximum				
	year-to-year		trend			year-to-year		trend			year-to-year		trend		
	mean	s.e.	Δ	*β*	s.e. (*β*)	mean	s.e.	Δ	*β*	s.e. (*β*)	mean	s.e.	Δ	*β*	s.e. (*β*)
April	−0.5	0.20	1.9	0.050	0.017	1.4	0.23	2.6	0.069	0.018	2.1	0.34	3.8	0.101	0.026
May	4.1	0.19	2.0	0.052	0.015	9.8	0.22	0.7	0.019	0.020	15.7	0.27	0.5	0.012	0.025
June	8.4	0.20	1.8	0.047	0.017	13.8	0.24	0.9	0.022	0.021	19.4	0.30	0.9	0.024	0.027
															
CAP 4-w pre-inc.	0.6	0.19	1.5	0.041	0.016	5.6	0.21	1.7	0.045	0.018	11.3	0.28	2.5	0.066	0.023
CAP incubating	5.1	0.20	0.7	0.019	0.018	10.7	0.24	−0.8	−0.021	0.022	16.6	0.29	−1.3	−0.033	0.026
CAP 4-w post-hatch	8.6	0.22	1.7	0.045	0.018	13.9	0.25	1.1	0.028	0.023	19.5	0.32	1.3	0.034	0.029
															
BG 4-w pre-inc.	1.6	0.24	1.5	0.039	0.021	7.0	0.25	1.4	0.036	0.023	13.0	0.33	2.0	0.052	0.029
BG incubating	6.1	0.24	0.4	0.011	0.022	11.6	0.28	−0.6	−0.016	0.026	17.4	0.33	−0.7	−0.019	0.031
BG 4-w post-hatch	9.1	0.22	2.5	0.066	0.017	14.3	0.24	1.3	0.034	0.021	19.9	0.29	1.3	0.034	0.026

In a series of regressions, we examined the annual relationships between breeding success and temperature in 13 time periods throughout the breeding season. As several successive periods were highly correlated, we restricted the results to the following four sub-periods: eight weeks pre-hatching, subdivided into four weeks pre-incubating and incubating periods, plus four weeks post-hatching (table 2*a*). Overall, breeding success increased with warmer springs, but the effects of rising temperature differed somewhat between the species. In capercaillie, breeding success increased significantly with the minimum temperature during eight weeks pre-hatching and four weeks post-hatching. Broken down to the two contributing components, brood frequency increased with increasing temperature both during the pre- and post-hatch periods, whereas brood size only increased with temperature post-hatching. In black grouse, breeding success increased only with the minimum temperature during four weeks post-hatching, seemingly caused by a significant correlation between brood size and the minimum temperature during that period. When comparing the effects of rising temperature on the demographic traits between the two species, statistically significant differences were discernible only for brood frequency during pre-hatch periods (table 2*a*): whereas brood frequency increased markedly with temperature in capercaillie (*β*: 0.049; 0.068), this was not the case in black grouse (*β*: −0.001; −0.012).

**Table 2.** (a) Regressions between breeding success, brood frequency, and brood size and minimum temperatures pre- and post-hatching in capercaillie and black grouse. (b) Partial regressions after controlling for the effects of the variables ‘Small rodents’ and ‘Fox’. Diff. *β* denotes interspecific difference. (Statistically significant (*p* < 0.05) and near-significant (*p* = 0.05–0.10) values are in bold and italic, respectively.)

		capercaillie			black grouse			diff. *β*	
(a) regressions		*β*	*r*	*p*	*β*	*r*	*p*	*t*	*p*
breeding success	8 weeks pre-hatching	**0.188**	**0.36**	**0.03**	0.041	0.07	0.66	1.17	0.24
	4 weeks pre-incubating	0.097	0.25	0.12	0.040	0.11	0.50	0.66	0.51
	incubating	0.097	0.26	0.11	−0.010	−0.03	0.87	1.26	0.21
	4 weeks post-hatch	**0.150**	**0.44**	**0.005**	**0.146**	**0.38**	**0.02**	0.05	0.96
brood frequency	8 weeks pre-hatching	**0.068**	**0.43**	**0.007**	−0.012	−0.07	0.66	**2.27**	**0.03**
	4 weeks pre-incubating	**0.049**	**0.42**	**0.008**	−0.001	−0.01	0.95	**2.07**	**0.04**
	incubating	0.022	0.20	0.23	−0.008	−0.09	0.60	1.25	0.22
	4 weeks post-hatching	**0.035**	**0.34**	**0.04**	0.021	0.20	0.22	0.57	0.57
brood size	8 weeks pre-hatching	0.007	0.01	0.94	0.120	0.21	0.21	0.81	0.42
	4 weeks pre-incubating	−0.060	−0.13	0.43	0.079	0.22	0.19	1.46	0.15
	incubating	0.064	0.15	0.37	0.013	0.04	0.83	0.55	0.58
	4 weeks post-hatching	**0.136**	**0.34**	**0.04**	**0.138**	**0.35**	**0.03**	0.02	0.98
**(b) partial regressions**		***β*_p_**	***r*_p_**	***p***	***β*_p_**	***r*_p_**	***p***		
breeding success	8 weeks pre-hatching	*0.155*	*0.30*	*0.06*	0.040	0.07	0.65		
	4 weeks pre-incubating	0.090	0.24	0.14	0.018	0.05	0.73		
	incubating	0.088	0.24	0.18	0.016	0.04	0.81		
	4 weeks post-hatching	**0.115**	**0.34**	**0.03**	0.093	0.24	0.11		
brood frequency	8 weeks pre-hatching	**0.061**	**0.38**	**0.01**	−0.016	−0.10	0.54		
	4 weeks pre-incubating	**0.048**	**0.42**	**0.008**	−0.005	−0.05	0.77		
	incubating	0.019	0.18	0.33	−0.010	−0.10	0.60		
	4 weeks post-hatching	0.024	0.23	0.16	0.010	0.10	0.57		
Brood size	8 weeks pre-hatching	−0.020	−0.03	0.87	0.14	0.25	0.13		
	4 weeks pre-incubating	−0.076	−0.17	0.32	0.060	0.17	0.30		
	incubating	0.064	0.15	0.43	0.064	0.18	0.35		
	4 weeks post-hatching	*0.117*	*0.29*	*0.09*	*0.108*	*0.28*	*0.10*		

**Figure 2.**
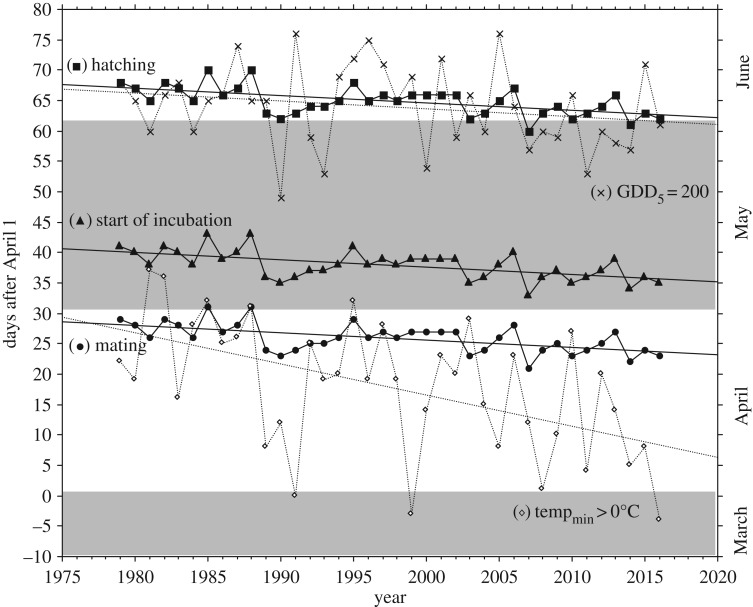
Arrival of spring (date of frost-free nights), arrival of summer (date of GDD_5_ = 200), and date of mating, start of incubation, and hatching in capercaillie during 1979–2016. In black grouse dates of peak of mating, start of incubation, and hatching occurred 5, 8, and 6 days after capercaillie.

**Figure 3.**
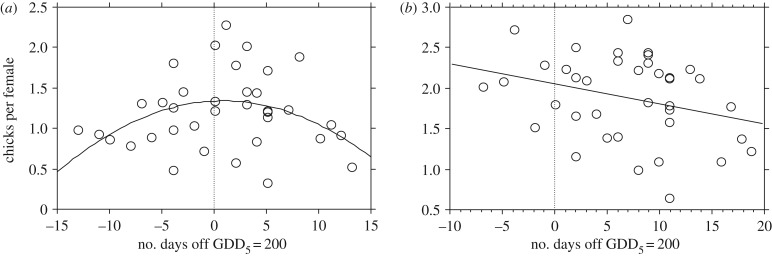
Breeding success (chicks per female) of capercaillie (*a*) and black grouse (*b*) plotted against how many days that hatching time was off the date of GDD_5_ = 200 (hatched vertical line). Early and late hatching are negative and positive values, respectively. In capercaillie, the best fitted line was a second-order polynomial function: *y* = 1.31 + 0.005 *x* − 0.004 *x*^2^; *R*^2^ = 0.15; *p* = 0.02. Black grouse was best fitted with a first-order function: *y* = 2.075 − 0.025 *x*; *R*^2^ = 0.09; *p* = 0.06.

The increase in April temperature caused the snow to disappear earlier; snow-free ground occurred 17–18 days earlier (*β* = −0.46, s.e. = 0.161), and the date of frost-free nights occurred 19–20 days earlier (*β* = −0.51, s.e. = 0.137), thus providing earlier access to ground foods before mating. However, both species responded little to earlier springs; mating time (and subsequent hatching time) advanced only 4–5 days throughout the study period (*β* = −0.12, s.e. = 0.029, *r* = −0.57, *p* < 0.001, figure 2). Because May and June temperatures increased less markedly, the onset of summer season (as indicated by a GDD_5_ of 200) only advanced 4–5 days, coincidentally paralleling the trend lines of hatching time in capercaillie and black grouse but with markedly larger year-to-year variation (*β* = −0.129, s.e. = 0.099, figure 2). Notably, the breeding success of both species was highest in years when hatching occurred around the time of GDD_5_ 200, i.e. in years when hatching ‘matched’ the date when bilberry shoot growth was completed (figure 3).

In Fennoscandia, predation on eggs and chicks varies with cyclic small rodents. This also applied to our study; hence, the positive effects of warmer springs might instead have been due to changes in rodent cycles or numbers of key predators. We therefore inspected the partial contribution of warmer temperatures after controlling for small rodent and red fox abundances (table 2*b*). In both species only minor changes occurred, *viz*. the relationship between brood frequency of capercaillie and minimum temperature four weeks after hatching was no longer statistically significant but still positive (*p* = 0.16). In black grouse, breeding success and brood size were no longer significantly correlated with minimum temperature four weeks post-hatching, but the tendencies were also here still positive (*p* = 0.11 and 0.10, respectively). The number of small rodents correlated with the summer temperature in the same year (*β* = 0.22, *r* = 0.38, *p* = 0.02), and fox abundances increased the year after rodent peaks (*β* = 0.078, *r* = 0.34, *p* = 0.04). We also examined if there was any relationship between the production of fledged chicks and summer temperatures 1 or 2 years earlier. No correlations were found in any of the two species (*R*^2^ < 0.03, *p* > 0.3, both).

## 5. Discussion

During the 38-year period, spring temperatures increased and so did the breeding success of both species. Closer analyses of annual variations showed a significant positive relationship between breeding success and temperatures during the whole breeding season (one month pre-laying to eight weeks post-hatching). No long-term trend was detected in the precipitation pattern in the breeding season, and no relationship was detected between breeding success and precipitation after hatching during a 30-year period [17]. In both species, the peak of mating (in late April) advanced 4–5 days during the study period. April was also the period when the temperature rise was most pronounced. Hence, to some extent, the temporal mating skew supports the general notion that mating in birds is triggered by rising temperatures [31] and advanced plant phenology [32]. However, because the onset of spring has advanced almost three weeks, the rather short forward skew of mating time reflects that breeding in northern latitudes is more closely controlled by the photoperiod [33,34]. As opposed to the pronounced rise in early spring temperatures, the temperature at hatching time in early June changed little. Hence, the small forward skew at hatching means that the chicks were exposed to about the same temperature as if the time of mating had remained constant.

From analysing the two demographic components that determine chick production separately, our results indicate that an increase in brood frequency may have contributed relatively more to the increasing breeding success than an increase in the size of broods in capercaillie. Brood frequency was the only trait that differed significantly between the two species, as it was strongly influenced by warmer springs during pre-hatching periods in capercaillie but not in black grouse. This again suggests that reduced egg loss—possibly combined with more yearlings breeding—rather than higher survival of hatched chicks has been most influential. We do not have information on nest predation in recent years, but during the first part of the study period, nest predation of capercaillie was quite high, averaging more than 50% annually during the 1980s [35]. During that time, the main nest predators (red fox and pine marten) were quite abundant [17]. The earlier ‘green-up’ due to the advancement of spring may have increased the availability of various food sources like small mammals, amphibians, and remains from winter kills for generalist predators, thereby lessening the pressure on grouse eggs. Simultaneously, the longer pre-hatching period with emerging new plant growth provided more cover and nutritive foods for females prior to mating and during incubation. For instance, the bog cottongrass, *Eriophorum vaginatum*—an important ground food in April—sprouted 10–14 days earlier in later years compared to the early 1980s (unpublished material). Presumably, the earlier access to nutritious ground foods improved the physical condition of females, shortening their need for recesses and exposure to nest predators.

The difference between the two species is difficult to explain. Black grouse females are about half the size of capercaillie females. They nest a few days later, nests are better camouflaged, females sit tighter on the eggs during incubation, and nest loss is lower than in capercaillie [35]. Thus, incubating black grouse may be less sensitive than capercaillie to changes in predation pressure. In addition, the tendency of higher survival of chicks in recent years might be related to better adult nutrition prior to hatching due to advanced plant phenology [15].

The predicted negative effect on breeding performance caused by asymmetrical temperature rise during the pre- and post-hatching periods [12] was not supported by our results. Although there was a skew towards earlier mating in warmer springs in our study (in magnitude similar to the results reported in the Finnish study), this skew was small compared to the much earlier snow-free ground and associated effects on plant phenology. In a previous study, Wegge *et al*. [13] reported that an earlier spring had little impact on the timing of peak caterpillar abundance in early summer. As the peak of geometrid and sawfly larvae stretched over more than 10 days [13], this ‘mismatch’ due to earlier hatching must have been negligible and not created any food shortage. Instead, the discrepancy between the results in our study and that reported from Finland is most probably due to different trends in the early summer temperatures: in the Finnish study—spanning an earlier time period than ours—post-hatching temperature decreased, leading to chicks hatching in slightly colder weather than ‘normal’, whereas in our study, summer temperatures changed very little.

Neither do our results support the prediction that forest grouse reproduction should decline after warm summers due to lower food quality mediated through changes in the chemical deterrents in the bilberry food plant [14]. Our analyses showed no relationship between summer temperatures 1 or 2 years earlier in either of the two species, as was the basis for this prediction. If the nutritive quality of chick food indeed is reduced by preceding high summer temperatures, then such an effect must have been minimal and certainly has not overridden other determinants of breeding success. In our study, not only the breeding success of grouse but also the abundance of small rodents correlated positively with summer temperature the same year. Presumably, the predation rate on eggs and chicks was then relaxed, leading to better breeding success. Hence, a negative relationship between preceding summer temperatures and breeding success, as inferred by Selås *et al*. [14], might instead have been caused by changing predation rates related to the vole cycle.

In the Scottish study [15], declining capercaillie breeding success during 1975–1999 correlated with a lower rise in temperature during the month of April due to a cooling period in the middle of the month. Poorer nutrition before incubation was offered as a plausible explanation. Contrasting the situation in Scotland—possibly due to different time periods—April temperatures rose markedly in our study, correlating with better breeding. However, in both studies, the effects on the physical condition of females prior to mating and incubation might have been a causal factor for the contrasting results. The study of black grouse in the French Alps [16] found no long-term effects on breeding success from changing winter and summer climate measured by the North Atlantic Oscillations; only short-term effects of extreme weather were detected. Possible impacts of changing spring weather were not assessed, but no declining trend in productivity was detected during the 17-year study period.

The effects of warmer springs—and climate change in general—probably affect the breeding performance of larger, mono-brooding species differently than the smaller, multi-brooding species. In the former, the total time from mating until brood independence is about four months, whereas passerine nestlings fledge *ca* 1.5 months after mating. Although grouse may re-nest if the first nest is robbed [36], these species have limited opportunities to repair a failed breeding attempt. Passerines, on the contrary, have multiple such attempts and also raise more than one brood during favourable conditions. Logically, therefore, a mismatch in the larger mono-brooding species like grouse is likely to influence the breeding success more negatively than among the smaller passerines.

Caterpillars are closely linked to their host plants, and hatching and irruption normally occur in synchrony with bud burst and sprouting of new leaves [37,38]. For insectivorous birds, warmer springs may have adverse effects on their breeding due to phenological mismatches at one or two successive stages. Firstly, and as shown by Visser & Holleman [39], the caterpillars may erupt before the host plant/tree has started new leaf growth, leading to subsequent die-offs of larval food before the birds hatch. Secondly, even if the phenology of both host and caterpillars match, both may advance more than the time of bird mating [9]. There is recent evidence that the first type of mismatch—the one between host plant and caterpillar hatching—may over time be ‘repaired’ by adaptation; in the winter moth *Operopthera brumata*, hatching too early was reduced by site-specific selection of successful, late-hatching eggs [40]. So far, adaptation among birds for earlier breeding to match the temporal advancement in food has mostly been dealt with on theoretical grounds [10,41]. As breeding in birds, at least in mono-breeding species, is more strictly regulated by the photoperiod [34,42], warmer springs are likely to advance the temporal occurrence of invertebrate foods more than the onset of bird breeding, at least in a short-time perspective.

As a larger mono-brooding species with the time of breeding strongly regulated by the photoperiod, we would expect boreal forest grouse to be adversely affected by warmer springs. Our results do not support this prediction; instead we show that reproductive output was enhanced—not reduced—in earlier and warmer springs. Why this contradictory result? So far, the extent of temporal mismatching caused by warmer springs—asynchrony between hatching time and optimum food sources and/or weather conditions for chicks—has not been critically examined for boreal grouse. In our study, breeding success was highest when the birds hatched around the time when bilberry shoot growth was completed, indicated by a GDD_5_ of 200. In the boreal forests, bilberry is the main host plant of the caterpillars needed by the newly hatched chicks [19–21,43]. The preliminary study by Wegge *et al*. [13], the first to measure the phenology of caterpillars in bilberry-dominated vegetation, found little temporal difference in the timing of the combined geometrid and sawfly caterpillar peak between a warm spring with early grouse breeding and a colder spring with late breeding; in both years the caterpillar peak lasted nearly two weeks. In northern coniferous forests, snow covers the ground in early April, which might preclude rising temperatures in April from stimulating new growth in bilberry, thereby maintaining closer synchrony between the emergence of caterpillars and new growth of their host plant.

If the temporal relationships between caterpillar abundance and spring weather remain more or less unaltered in bilberry habitats, warmer springs may not adversely affect the availability of food for the chicks. Also, and in contrast with the study of black grouse breeding in Finland [12], the temperature during hatching in early June is increasing, albeit much less than during the pre-hatching periods. From the influences of earlier and warmer springs on vegetation and breeding phenology—as reported in this study—we therefore predict that breeding success will not be adversely affected; warmer springs may even enhance the breeding performance of boreal forest grouse. However, because the caterpillar food source plays a crucial role in the breeding performance of these species, the temporal relationships between bilberry leafing and caterpillars—and how they vary with spring temperatures—need to be thoroughly examined.

Summing up, our study does not lend evidence to the general notion that warmer climate will adversely affect the reproduction of birds and animals in northern latitudes [6]. Instead, we found that the reproductive output of two species of boreal forest grouse increased along with warmer and earlier springs. However, our study only assessed the impact on breeding performance. Through effects on environmental variables, a warmer climate may have adverse effects on other demographic processes, for instance on the survival of adult birds, thereby dampening or nullifying the positive effects on reproductive output. A recent study of the same populations [18] showed that, in spite of increasing trends in breeding success, the population sizes of both species remained more or less constant. As pointed out by Stenseth & Mysterud [44] and Winkler *et al*. [45], climate change—with its impact on phenology—is likely to affect several life-history traits and therefore calls for an all-embracing examination in order to identify critical relationships.

## Data accessibility

Background bird census and climate data files available from the Dryad Digital Repository [46]: https://doi.org/10.5061/dryad.g0295.
